# Investigation of Parallel Radiofrequency Transmission for the Reduction of Heating in Long Conductive Leads in 3 Tesla Magnetic Resonance Imaging

**DOI:** 10.1371/journal.pone.0134379

**Published:** 2015-08-03

**Authors:** Clare E. McElcheran, Benson Yang, Kevan J. T. Anderson, Laleh Golenstani-Rad, Simon J. Graham

**Affiliations:** 1 Physical Sciences Platform, Sunnybrook Health Sciences Institute, Toronto, Ontario, Canada; 2 Department of Medical Biophysics, University of Toronto, Toronto, Ontario, Canada; 3 Massachusetts General Hospital, Harvard Medical School, Charlestown, Massachusetts, United States of America; Brandeis University, UNITED STATES

## Abstract

Deep Brain Stimulation (DBS) is increasingly used to treat a variety of brain diseases by sending electrical impulses to deep brain nuclei through long, electrically conductive leads. Magnetic resonance imaging (MRI) of patients pre- and post-implantation is desirable to target and position the implant, to evaluate possible side-effects and to examine DBS patients who have other health conditions. Although MRI is the preferred modality for pre-operative planning, MRI post-implantation is limited due to the risk of high local power deposition, and therefore tissue heating, at the tip of the lead. The localized power deposition arises from currents induced in the leads caused by coupling with the radiofrequency (RF) transmission field during imaging. In the present work, parallel RF transmission (pTx) is used to tailor the RF electric field to suppress coupling effects. Electromagnetic simulations were performed for three pTx coil configurations with 2, 4, and 8-elements, respectively. Optimal input voltages to minimize coupling, while maintaining RF magnetic field homogeneity, were determined for all configurations using a Nelder-Mead optimization algorithm. Resulting electric and magnetic fields were compared to that of a 16-rung birdcage coil. Experimental validation was performed with a custom-built 4-element pTx coil. In simulation, 95-99% reduction of the electric field at the tip of the lead was observed between the various pTx coil configurations and the birdcage coil. Maximal reduction in E-field was obtained with the 8-element pTx coil. Magnetic field homogeneity was comparable to the birdcage coil for the 4- and 8-element pTx configurations. In experiment, a temperature increase of 2±0.15°C was observed at the tip of the wire using the birdcage coil, whereas negligible increase (0.2±0.15°C) was observed with the optimized pTx system. Although further research is required, these initial results suggest that the concept of optimizing pTx to reduce DBS heating effects holds considerable promise.

## Introduction

Magnetic resonance imaging (MRI) is well known to provide images with superb signal contrast between various soft tissues within the human body, especially for the brain. Thus, MRI is the modality of choice for pre-operative and post-operative assessments involving placement of deep brain stimulation (DBS) implants [1]. DBS implants send electrical impulses to deep brain nuclei to treat neurological disorders such as Parkinson’s disease, essential tremor, primary dystonia and obsessive compulsive disorder [2]. The DBS implant must be placed precisely, as maximum stimulation of the target region is required for effective treatment, whereas inadvertent stimulation of regions adjacent to the target can cause side effects such as muscle contractions, ocular deviations, headache and depression [3]. With the increased availability of high field MRI at 3 T over the last decade, the associated gain in tissue contrast in deep brain nuclei has created the potential for physicians to improve DBS targeting and placement [4].

Although 3 T MRI is advantageous pre-operatively, its use post-operatively in DBS patients is hampered by substantial safety concerns. The long implanted leads intrinsic to DBS are susceptible to localized heating effects from the radiofrequency (RF) transmission field that is used for resonant excitation of tissue magnetization during MRI [5, 6]. The electric component (E-field) of the RF field deposits energy in the patient during MRI, potentially causing damage due to tissue heating [7]. This energy deposition can be quantified in terms of a power metric, the specific absorption rate (SAR), spatially averaged over the entire subject (global SAR) or over a small region of specific mass such as 1 g or 10 g (local SAR). For the case of 1 g SAR,

$$SAR_{1g}(t)=\int_{1g}\frac{\sigma (\vec{r}){\left|\vec{E}(\vec{r},t)\right|}^{2}}{\rho (\vec{r})}dv$$ (1)

where *σ* is the conductivity of the medium, ρ is the density, and $\left|\vec{E}(\vec{r},t)\right|$ is the magnitude of the E-field at position $\vec{r}$ and time *t*. The integral is performed over the volume of interest, in the case of 1 g SAR, over a volume with mass of 1 g.

In patients without metallic implants, global SAR is a useful measure of energy deposition because the E-field varies slowly in space. Limits are set on clinical MRI systems to ensure adherence to global SAR levels as required by regulatory agencies, ensuring that elevations in tissue temperature are safely constrained during routine imaging. However, implanted devices produce small regions of high E-field leading to large local SAR, which can result in large temperature increases near the tips of the implanted leads [8]. Across a number of studies, highly variable heating effects have been observed [5, 9] with complex dependence on factors such as scan parameters, MRI system, static magnetic field, lead location, lead design, lead length, RF coil design and pulse sequence. The level of heating is not directly related to whole-body averaged SAR [10, 11]. Furthermore, clinical MRI systems currently lack a mechanism for monitoring and safely regulating local SAR or local temperature increases.

Previous work in mitigating localized heating in patients with implanted devices falls into three categories: (a) device modifications that minimize coupling with the RF field; (b) monitoring the current induced by RF coupling; and (c) RF transmit coil modifications [12–20]. In the first category, there have been many attempts at modifying implants to reduce RF coupling, particularly relating to intravascular guide-wires for interventional MRI [12–16]. Although gains can be made by such approaches, they are not “backward-compatible” with existing implant designs. In the second category, non-invasive monitoring of heating *in vivo* is an important step towards improving safety in a clinical setting. Current in the implant can be monitored by an external sensor [17], or by RF receiver coils of the MRI system [18]. These methods provide excellent tools to monitor heating surrounding implanted leads for safety *in vivo*, as part of solving the lead heating problem.

In the third category, early promising work has been undertaken to minimize heating in implanted leads by modifying RF transmit coils. Eryaman *et al*. [21] introduced a method to modify the inputs of a birdcage coil to produce a plane of zero E-field, minimizing heating in implants located in that plane while maintaining B_1_-field homogeneity. This idea was subsequently extended to create null planes of arbitrary orientation, providing more flexibility to accommodate patient and implant positioning [22]. In practice, this method may have limitations due to the model used for understanding the spatial properties of the null region and determining input voltages, which assumes ideal uniform cylindrical media of infinite length regardless of the subject, whereas the situation is more complex in reality. In addition, implementation results in higher global SAR compared to the use of a standard birdcage coil for head imaging with no implants present.

Other coil modifications are also of substantial interest, especially those exploiting the concept of parallel RF transmission (pTx). Using pTx, multiple RF coils are driven individually to produce the overall RF transmission field. The additional degrees of freedom provided by multiple input signals potentially provides greater control over the spatial distribution of the transmitted B_1_-field and E-field compared to what is achievable using quadrature excitation, the standard mode of RF transmission on MRI systems. Benefits of pTx have been demonstrated in multiple applications at high field strength (3 T and above), including improved B_1_-field homogeneity and spatially-selective excitation, reduced global SAR and scan times, reduced shading surrounding orthopaedic implants, and monitoring implant coupling [19, 23–28].

Early work, in experiments pertinent to cardiac MRI, and simulations pertinent to MRI of DBS implants [29, 30], has also shown the potential of pTx to produce an E-field distribution that substantially reduces the potential for localized heating from implanted devices. Etezadi-Amoli *et al*. [31] used various current monitoring devices to minimize RF coupling in implanted cardiac devices with a 4-element planar pTx array. Eryaman *et al*. [29] investigated use of an 8-channel pTx head coil to minimize heating in DBS leads through numerical simulation. Both implementations utilized independent RF inputs and amplifiers for each coil element. Although flexible, such pTx methodology comes with substantial experimental complexity and associated cost.

The present work studies pTx concepts further, focusing on future application to DBS, in the simpler case where RF inputs to coil elements differ only in terms of their amplitude and phase. Numerical simulations and experimental validation are used to investigate static amplitude and phase variation to reduce heating in long implanted wires. Numerical simulations and cost function optimization are used to investigate the utility of pTx to reduce power deposition at the tip of an implanted wire in a uniform, tissue-mimicking medium, including a localized, volumetric constraint to maintain RF magnetic field homogeneity. Three pTx coil configurations (two, four and eight element) are studied to determine the extent of power reduction in each case, as well as coil performance characterized by B_1_-field homogeneity, compared to the performance of a conventional transmit/receive birdcage coil. The validity of the numerical simulations and pTx benefits are also assessed in proof-of-concept MRI experiments conducted at 3 T.

## Materials and Methods

**Fig 1:**
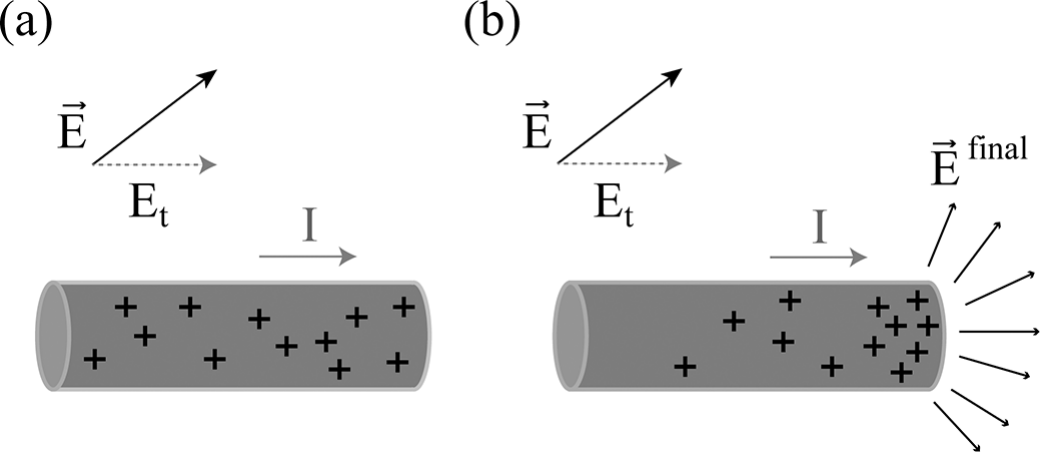
Electric field coupling with wire. Coupling of the electric field ($\vec{E}$) with the wire (shown in grey). Charge distribution is represented by “+”. (a) The tangential component of $\vec{E}$, *E*_*t*_, induces a current (*I*) along the wire. (b) The current produces a charge build-up at the tip of the wire creating a concentrated electric field (${\vec{E}}^{final}$), and enhancing the overall power deposition at the tip of the wire according to Ohm’s law.

Heating of long implanted wires (leads) during MRI has been characterized through numerous safety studies involving numerical simulations and experiments [32–38]. Biophysically, the effect is well described by modelling the lead as a transmission line [39, 40]. The lead is excited as an antenna by the tangential component of an external E-field, *E*_*t*_, created by the RF transmit coils. A current, or propagating electromagnetic field, is induced which terminates at the tip of the lead, resulting in charge build-up. The charge build-up in turn causes a higher E-field (*E* ^*final*^), which, through resistive heating, translates to increased power deposition (Fig 1). In this case, Eq 1 can be used to calculate local SAR from *E* ^*final*^, with volumetric integration over 1g of tissue appropriately localized at the lead tip. Temperature increase can then be related to the power deposited due to the RF source, as modelled by the Pennes bioheat equation [41].

If the wire is modelled as a transmission line with distributed impedance and admittance, then the induced current can be estimated from the effects of a distributed voltage source which is related to *E*_*t*_ [42]. The extent of charge build-up at the tip of the wire (and thus power deposition) will depend on the spatial dependence of *E*_*t*_ along the wire. Thus, to minimize charge build-up, a method is required for manipulating the spatial distribution of the E-field that is generated in RF transmit coils.

pTx has potential to fulfill this need. The combination of multiple sources with varying amplitude and phase allows for the manipulation of the coherence of fields (B_1_-field and E-field) generated by each RF coil at the center of the subject. Previously, this concept has been exploited to produce a highly homogeneous B_1_-field by ensuring that the fields produced by opposite elements are coherent. There are multiple RF inputs to the *N*-channels used in pTx that will produce a uniform B_1_-field [43]. Current pTx sequence design includes global SAR limits as a constraint or as a term in the optimization function to ensure no regions of high E-field are created [44, 45]. Useful, homogeneous B_1_-fields are obtained in the presence of these constraints. Such applications are largely indifferent to the precise nature of E-field distribution, except that minimizing global energy deposition is of interest. This implies that the E-field distribution can be varied with pTx and that sufficient B_1_-field homogeneity can be produced through numerous solutions, introducing the possibility to tailor the E-field to a desired pattern, such as a spatial distribution that minimizes coupling with an implanted wire.

### Numerical Simulations

A commercially-available Method of Moments (MoM) solver, (FEKO, Altair, MI, USA), was used to develop electromagnetic simulations of RF transmission for different pTx and conventional coil configurations that enclosed a uniform finite cylindrical medium representing the head, and that included a simple approximation of a DBS implant (a straight wire). The “static RF shimming” version of pTx was adopted: *ie*. use of a common RF pulse shape but different amplitude and phase for each transmit channel. Although less flexible than dynamic shimming (where amplitude and phase are allowed to vary independently with time during RF transmission for each channel), static RF shimming was chosen for its ease-of-implementation (in both simulation and experiment) and was judged to be sufficient for establishing proof-of-concept. Initial conditions were chosen for “quadrature mode” RF shim inputs that produced optimal B_1_-field homogeneity: equal amplitude for each channel, and phase variation of 360°/*N* where *N* was the number of coil elements in a given pTx configuration.

For each pTx coil configuration, the Nelder-Mead Simplex optimization algorithm [46] from the built-in optimization function of FEKO was used to manipulate the amplitude and phase applied to each RF channel. The cost function for optimization contained two terms promoting E-field minimization and B_1_-field homogeneity:

$$\text{min}\;\left({\left\{\left|\vec{E}(\vec{r})\right|\right\}}_{{}^{ROM}}+\lambda {\left\{\frac{\underset{\vec{r}}{\text{max}}\;\left|{\vec{B}}_{1}\left(\vec{r}\right)\right|-\left|{\vec{B}}_{1}\left(\vec{r}\right)\right|}{\underset{\vec{r}}{\text{max}}\;\left|{\vec{B}}_{1}\left(\vec{r}\right)\right|}\right\}}_{POI}\right)$$ (2)

where $\left|\vec{E}(\vec{r})\right|$ is the magnitude of the E-field at position $\vec{r}$, $\left|{\vec{B}}_{1}(\vec{r})\right|$ is the magnitude of the RF magnetic field at position $\vec{r}$, $\vec{r}$ is the position relative to the center of the imaged object and λ is a weighting factor used to vary the relative importance of the B_1_-field homogeneity and the E-field minimization. The E-field and B_1_-field are optimized over a region of minimization (ROM) and pre-defined plane of interest (POI), respectively. According to the analytical and simulation models discussed above [39, 40], coupling between the E-field and the lead results in charge build-up at the tip of the lead, and thus the highest E-field at this location. An important scenario where this condition is met occurs when the value of *E*_*t*_ is small along the length of the lead. Therefore, appropriate terms were included in the cost function to explore both effects separately: a) minimizing the E-field at the tip of the lead; and b) minimizing the tangential E-field along the lead via an appropriate ROM. In the case of a straight wire oriented in the z-direction, the E-field is approximately parallel to the wire and thus the minimization of the tangential component of the E-field is approximated by minimization of the magnitude of the E-field.

**Fig 2:**
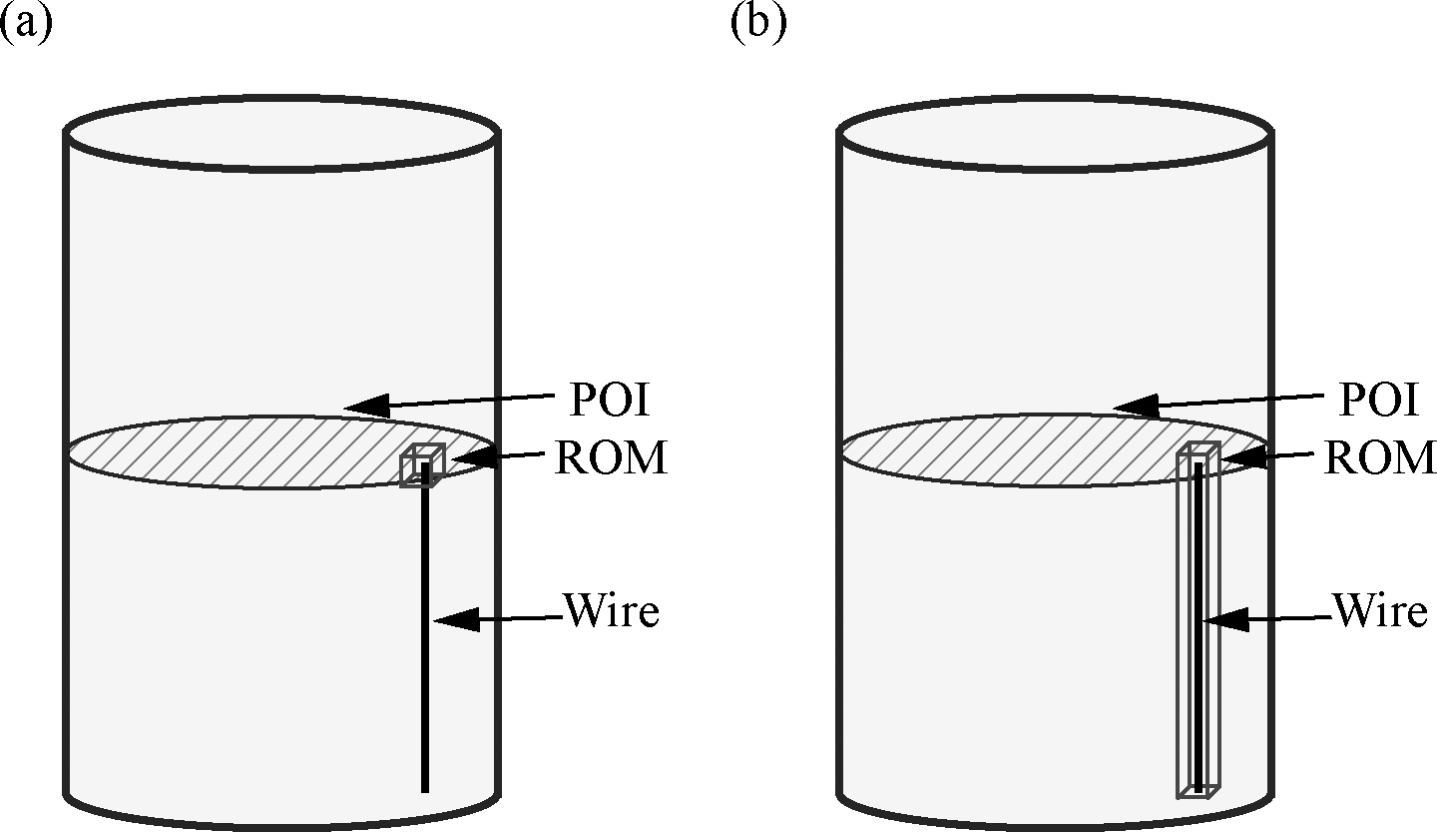
Geometry of test medium with optimization regions. Uniform finite cylindrical medium with straight, perfectly conducting wire used in numerical optimization. The plane of interest (POI) for monitoring B_1_-field homogeneity is shown shaded. The region of minimization (ROM) is shown in (a) for minimizing the E-field at the tip of the wire, and (b) along the length of the wire.

The second term in the optimization equation maximized the B_1_-field homogeneity. Throughout, the POI was oriented normal to the lead and positioned in the center of the medium. Fig 2 depicts the ROM and POI investigated in this work. Preliminary simulations determined that an even weighting of the two terms in the cost function (*λ* = 1) produced the best results and this λ value was used in all subsequent simulations.

### Coil Configurations

**Fig 3:**
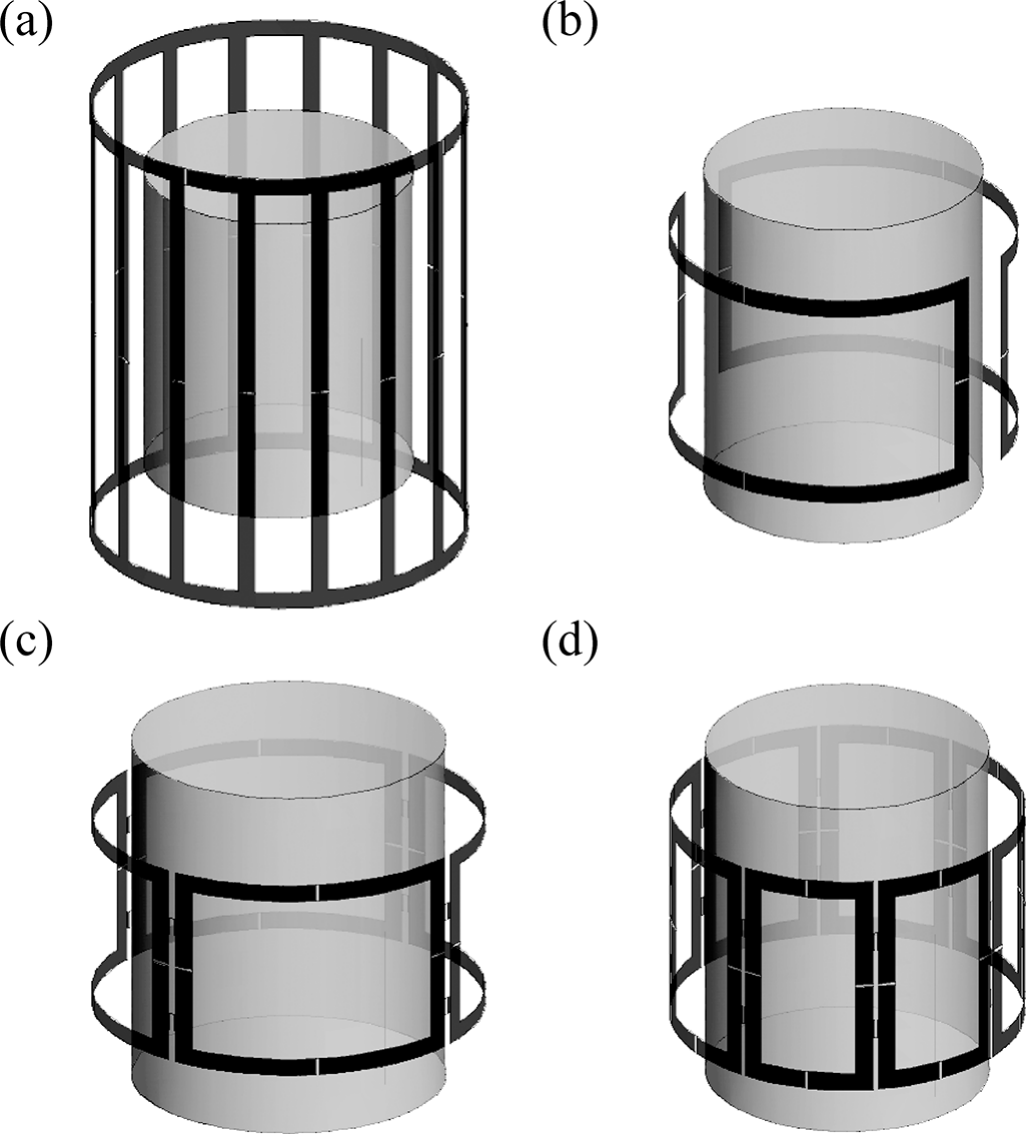
Coil configurations. Uniform cylindrical phantom with coil configurations for (a) 16-rung birdcage coil, (b) 2-element pTx, (c) 4-element pTx and (d) 8-element pTx systems.

To investigate the utility of pTx as a function of channel count, cylindrical configurations were investigated with *N* = 2, 4, and 8 (Fig 3) where *N* is the number of channels. For a given configuration, *N* coil elements were placed with equal angular spacing around the cylinder. Coil element size was determined for each configuration to maximize B_1_-field penetration and minimize B_1_-field inhomogeneity, when excited in quadrature mode. The coils were tuned, matched and decoupled via capacitive decoupling [47] between nearest neighboring elements. Tuning, matching and decoupling of all coils were done with the coil loaded with the uniform, cylindrical phantom with no wire present. Simulations were done to verify the validity of the tuning, matching and decoupling capacitor values with the wire present in various locations. Although the wire affected the tuning, matching and decoupling, no substantial degradations were observed. The pTx systems were compared to a standard 16-rung birdcage coil also operating in its quadrature mode. The input voltages for the birdcage coil were chosen such that the average B_1_-field from the birdcage coil was equivalent to the average B_1_-field from pTx in the imaging POI.

**Fig 4:**
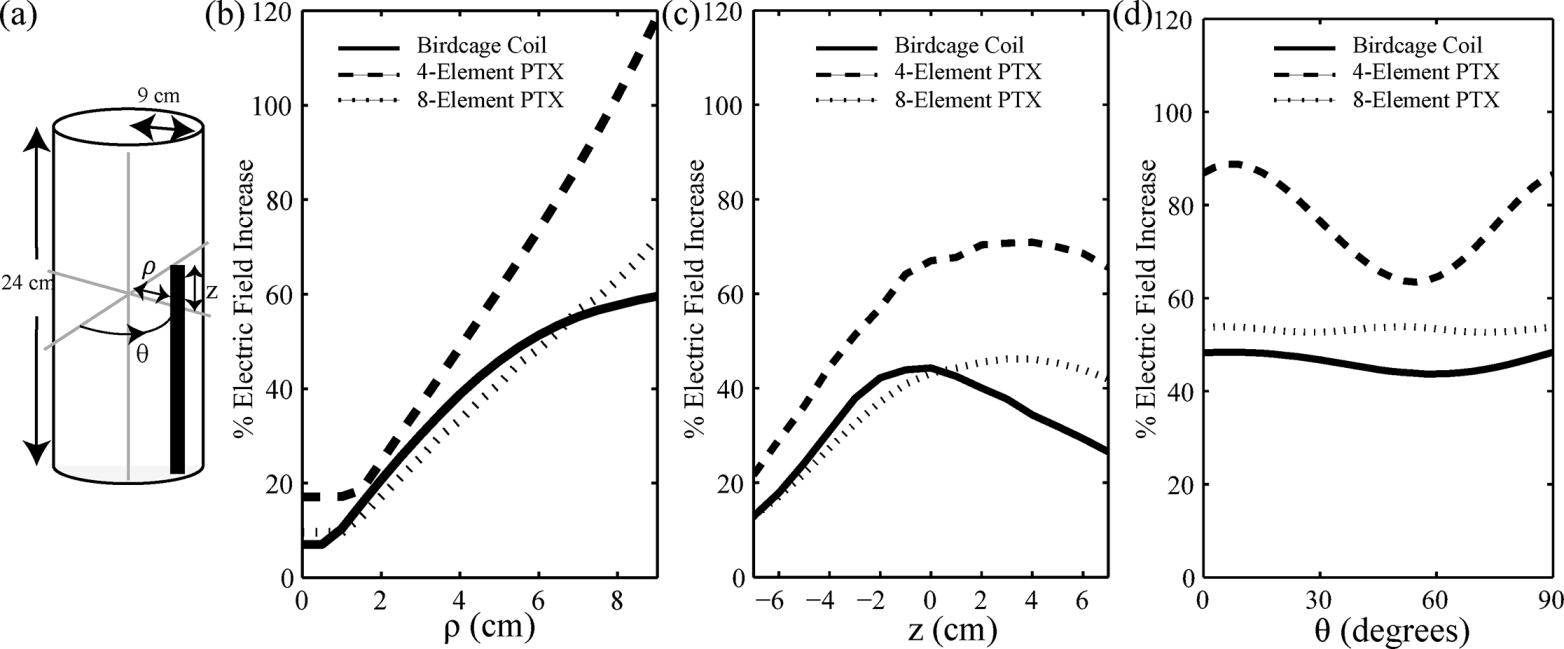
Electric field enhancement as function of wire position. (a) Uniform cylindrical medium with implanted wire. Radial position (ρ), position of wire tip (z) and angular location (θ) are indicated. (b) Preliminary simulation results showing the percentage E-field increase between wire tip and background as a function of ρ with z = 0 cm, θ = 90°. Both pTx configurations exhibited E-field increases that grew monotonically from zero as the wire was moved toward the edge of the medium, with 8-element pTx showing smaller effect than 4-element pTx. In contrast, the E-field increase for the birdcage coil grew from zero to a plateau at approximately ρ = 7 cm. (c) Percentage E-field increase between wire tip and background plotted as a function of z with ρ = 7 cm, θ = 90°. Maximal increase occurred between z = -1 cm and z = 4 cm for 4-element and 8-element pTx, respectively, whereas the birdcage coil produced maximal effect between z = -2 cm and z = 0 cm. (d) Percentage E-field increase between wire tip and background plotted as a function of θ with ρ = 7 cm, z = 0 cm. Maximal heating occurs at θ = 90° with 4-element pTx showing the largest angular variation.

To approximate the human head and an implanted DBS lead, a cylindrical uniform medium (9 cm radius, 24 cm length) was modelled with a permittivity (ϵ) of 80 and conductivity (σ) of 0.47 S/m, including a perfectly conducting metallic wire of 0.5 mm radius (Fig 4A). The wire was positioned in cylindrical coordinates parallel to the long (z) axis of the cylinder, offset radially from the center, and at angle θ, based on two criteria: substantial implant heating and realistic positioning for a DBS lead. To determine scenarios that produce significant heating, preliminary simulations were run with the wire terminating at different positions. This was done for three coil configurations: a birdcage coil with 16 rungs and pTx with *N* = 4 and *N* = 8, all operating in quadrature mode. The position of the wire was varied along the radial direction (ρ) with z = 0 cm, θ = 90°; in the z-direction with ρ = 7 cm, θ = 90°; and angularly (θ) with ρ = 7 cm, z = 0 cm. Two notable trends were observed with respect to wire positioning: higher coupling was observed as the wire approached the coil elements (by varying ρ), and higher coupling was seen at certain wire lengths corresponding to resonant effects [39, 40, 48, 49]. To demonstrate these trends in more detail, Fig 3B and 3C show the increase of E-field at the tip of the wire relative to minimum E-field magnitude within the medium plotted as a function of ρ and z, respectively. Along the radial direction, the E-field enhancement for both pTx configurations increased monotonically as the wire tip was moved from the center toward the edge of the medium, with a maximum observed closest to the coils. Compared to the *N* = 4 configuration, the *N* = 8 configuration produced less enhancement throughout. In the case of the birdcage coil, the enhancement reached a plateau at approximately ρ = 7.0 cm, with a slight decrease in slope as the wire tip approached the edge of the phantom. In the z-direction, both pTx systems showed maximal E-field enhancement for z ranging from approximately -1 cm to 4 cm, with the *N* = 8 configuration again showing less enhancement. Conversely, the birdcage coil showed maximal enhancement for z ranging from approximately -2 cm to 0 cm, with a sharper peak than seen in either pTx configuration. In the angular direction, minimal variation occurred for the 8-element pTx and the birdcage coil, although a small, periodic oscillation was observed with a 45° and 90° period, respectively. This agrees with the structural periodicity of the coils. The 4-element pTx showed a larger amplitude variation than the 8-element pTx and the birdcage coil with a 90° period, due to the larger coil elements. For subsequent simulations and experiments, the worst case heating scenario of θ = 90° was used.

In patients, DBS leads terminate deep in the brain, at various locations, depending on the disorder that is being treated, and loop around the back of the head from the point of insertion. Accordingly, and additionally informed by the simulations mentioned immediately above, important scenarios to simulate were judged to be when the wire terminated at various deep brain sites, near (but not precisely at) the central region (ρ = 2.5 cm) and at a mid-region (ρ = 6 cm), and when the wire was close to the edge of the phantom (ρ = 7.5 cm, ρ = 8 cm). Two z positions of the wire tip were examined: z = 0 cm and z = 2 cm, positions where the preliminary simulations showed maximum E-field enhancement in the 4-element and 8-element pTx configurations. In addition to evaluating our optimization algorithm for asymmetrical lead placement, positioning of the wire tip at z = 0 cm was evaluated to ensure that E-field minimization at the tip of the wire would not produce a void in the B_1_-field when the tip of the wire coincided with the imaging plane. The wire was positioned between coils 1 and 2 in the 2-element and 4-element configuration, and coils 2 and 3 in the 8-element configuration.

Using the optimization procedure described above, optimal amplitude and phase were estimated for each wire scenario and for each pTx configuration. The E-field and B_1_-field were subsequently calculated, including for a 16-rung birdcage coil. The inputs for each coil in the pTx configurations were weighted such that the average B_1_-field through the imaging plane was consistent across the various geometries.

### Experimental Validation

To assess the level of agreement between simulation and experiment, a customized 4-channel pTx system was built for use on a 3 T research-dedicated MRI system (MR750, GE Healthcare, Waukesha, WI). A 4-channel configuration was chosen as simulations showed that a 98% reduction of E-field was achieved near the wire tip with high magnetic field homogeneity (see Results) while minimizing the cost and complexity of implementation. The pTx system was designed in several iterative cycles, with hardware practicalities informing how simulations were conducted, and simulation results subsequently informing the final hardware set-up. In particular, the final set of pTx simulations determined that localized power deposition was optimally decreased by RF shim parameters that exhibited minimal amplitude variation across channels (see Table 1 below). Thus, the experimental setup was designed only with phase shimming capability (*ie*. without amplitude shimming). Supporting simulations were subsequently run to establish the optimal inputs for phase shimming only, which were then applied to the experimental system to compare simulation and experiment.

**Table 1:** Optimized amplitude and phase shifts applied to pTx channels.

	Relative Amplitude			Phase (°)		
Channel	pTx2	pTx4	pTx8	pTx2	pTx4	pTx8
ρ **= 2.5 cm, z = 0 cm**						
1	0.204	0.325	0.537	343.5	49.1	65.2
2	0.215	0.476	0.481	351.3	0.0	133.1
3	-	0.394	0.417	-	187.9	105.9
4	-	0.403	0.535	-	292.6	58.3
5	-	-	0.590	-	-	191.3
6	-	-	0.426	-	-	228.6
7	-	-	0.427	-	-	234.4
8	-	-	0.412	-	-	198.8
ρ **= 6 cm, z = 0 cm**						
1	0.504	0.976	0.372	0.3	97.9	52.5
2	0.558	1.000	0.465	357.7	93.3	110.9
3	-	0.977	0.297	-	286.9	10.8
4	-	0.953	0.533	-	30.1	111.6
5	-	-	0.417	-	-	232.8
6	-	-	0.488	-	-	290.9
7	-	-	0.557	-	-	266.3
8	-	-	0.603	-	-	255.6
ρ **= 7.5 cm, z = 0 cm**						
1	0.367	0.557	0.453	74.6	25.9	41.4
2	0.539	0.556	0.477	91.8	17.2	99.3
3	-	0.528	0.509	-	206.6	26.9
4	-	0.543	0.515	-	286.0	107.3
5	-	-	0.489	-	-	199.8
6	-	-	0.522	-	-	195.1
7	-	-	0.520	-	-	265.3
8	-	-	0.444	-	-	267.8
ρ **= 8 cm, z = 2 cm**						
1	0.333	0.415	0.521	72.2	120.2	53.6
2	0.544	0.422	0.052	94.6	117.1	143.8
3	-	0.502	0.543	-	349.7	13.0
4	-	0.614	0.516	-	0.0	144.6
5	-	-	0.428	-	-	250.6
6	-	-	0.529	-	-	278.7
7	-	-	0.063	-	-	357.9
8	-	-	0.620	-	-	295.1

The coil elements of the pTx system, which were designed to enable both RF transmission and MRI signal reception, were constructed as rectangular loop antennas of copper tape located at 90° intervals around a cylindrical acrylic tube. Each element was tuned, matched and decoupled via capacitive decoupling of nearest neighbors experimentally while the coil was loaded with a uniform, cylindrical phantom (described below). Once the coil fabrication was complete, the dimensions, geometry and capacitor values were used in the numerical simulations to ensure agreement between simulation and experiment. The RF transmit signal from the MRI system was split using a commercial Combiner/Divider (Werlatone Inc, New York, USA) to supply the four independent RF input signals. Optimized phase delays of φ_1_ = 40°, φ_2_ = 16°, φ_3_ = 200°, and φ_4_ = 301° were obtained via simulation and achieved using various lengths of coaxial cable to connect the outputs from the power divider to each coil element. Both transmit and receive chains followed the same path through the Combiner/Divider and thus both were phase shifted. Resultant images were reconstructed from a single quadrature receive channel, using the default capabilities of the MRI system.

The 4-channel pTx system was used to image a uniform phantom, which was constructed from an acrylic cylinder with inner diameter 18 cm and length 24 cm. The phantom was filled with polyacrylic acid (PAA) to simulate the permittivity and conductivity of brain tissue. A copper wire was fixed within the phantom with geometry similar to that shown in Fig 4, positioned at ρ = 6 cm, coinciding with simulation results, and with the tip of the wire at z = 0 cm. In addition, a fiber-optic MRI-compatible temperature probe (OpSens, Quebec City, Canada) was affixed to the tip of the wire to measure temperature elevations.

A fast recovery fast spin echo (FRFSE) pulse sequence (TR = 559 ms, TE = 100 ms, echo train length = 24, 1 slice, imaging time = 4:08) was used in heating experiments, across three coil configurations. A 10 min. cool-down period was implemented between each collection of imaging data. The first two configurations involved the pTx system in quadrature mode, and then in suppression mode, the latter mode determined by the optimized phase shifts from simulation results. The standard transmit/receive birdcage coil was subsequently imaged in standard quadrature mode for comparison. In each case, the RF amplitude, or "transmit gain", was based on automatic pre-scan of multiple subjects such that clinically relevant transmit gains were used for the birdcage coil and the pTx coils. Each data set was acquired twice to ensure reproducibility. The FRFSE sequence was chosen to provide sufficient power deposition to generate pronounced heating effects in un-optimized coil configurations and was not optimized for image quality. Image quality was assessed for the pTx system in suppression mode using a GRE sequence (TR = 2000 ms, TE = 40 ms, slice thickness = 10 mm, in-plane resolution = 256x256, FOV = 24 cm).

## Results

### Simulation

The optimal shim values for amplitude and phase for each wire placement and coil configuration are provided in Table 1. The amplitudes are unit-less weighting factors applied to each input voltage. Phase shifts are given in degrees. The standard deviation of amplitude variation across coils for a given simulation (wire placement and coil configuration) was less than 10%, excluding the 2-element coil configuration and the 8-element coil configuration specifically for ρ = 8 cm, z = 2 cm. In these exceptions, elements close to the wire showed attenuated amplitude.

**Fig 5:**
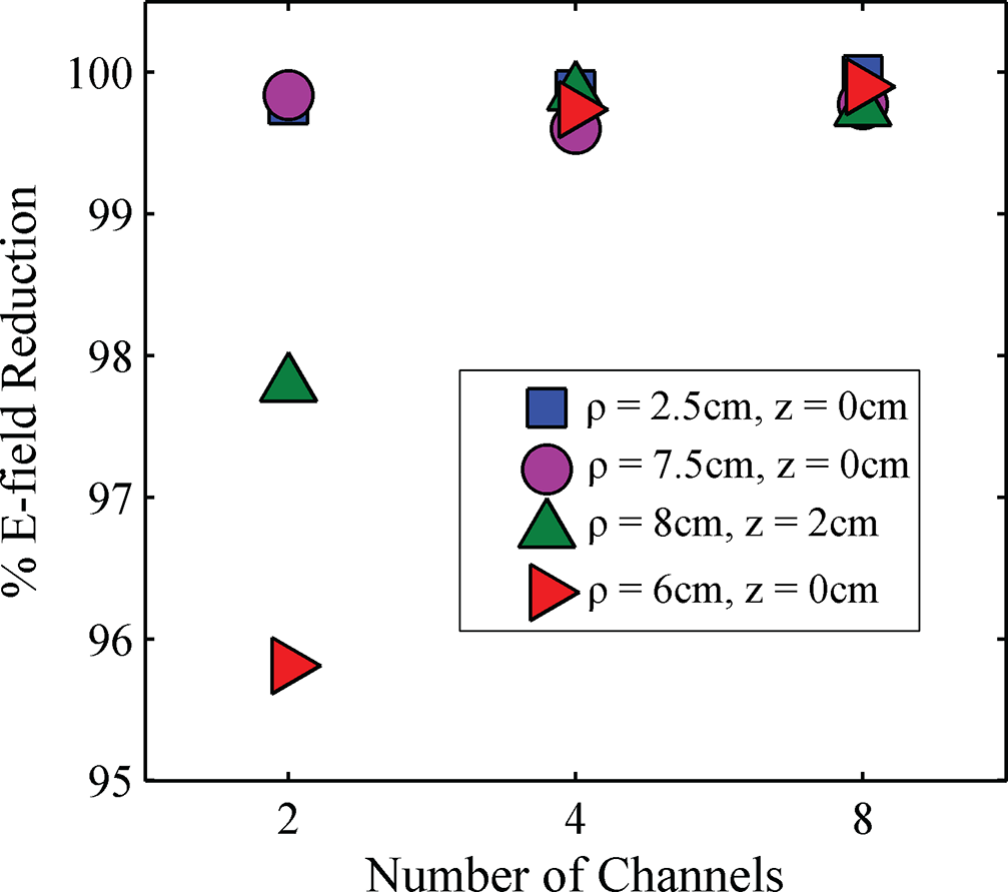
Percent reduction in E-field magnitude between pTx and birdcage coil for various pTx channel counts. Data for four wire positions are shown: ρ = 2.5 cm, z = 0 cm (blue squares); ρ = 7.5 cm, z = 0 cm (purple circles); ρ = 8 cm, z = 2 cm (green triangles); and ρ = 6 cm, z = 0 cm (red triangles).

Analyzing the effects of the simulated optimal shim values in greater detail, Fig 5 shows the extent that the E-field magnitude was decreased at the tip of the wire (the location of maximum E-field) versus *N*, the pTx channel count, for each wire location. The extent of decrease is given as a percentage, calculated with the corresponding E-field magnitude for the birdcage coil as a reference. An E-field decrease of at least 95% was found for all pTx channel counts and wire locations, with better performance obtained as *N* increased. The 4-channel and 8-channel pTx systems performed similarly with respect to this metric, with over 99.5% decrease in E-field for all wire positions in both configurations.

The US Food and Drug Administration (FDA) provides a limit of the ratio of 1g local SAR vs global SAR of 2.7 [50]. This ratio, denoted R, was calculated for the four coil geometries to contextualize the E-field decrease achieved by pTx in relation to use of the birdcage coil. To calculate R, peak local 1g SAR and global SAR were calculated for each wire location, with the local SAR evaluated at the same location as the peak local SAR in the birdcage coil (see Table 2 below). In the case of the birdcage coil, the R value was approximately 4 to 5 orders of magnitude above the FDA limit, whereas the R value was less than the FDA limit for all pTx geometries and wire locations. Furthermore, the R value was below 20% of the FDA limit in all but one case. In the case of 4-channel pTx for wire position ρ = 2.5 cm, z = 0 cm an R value of 2.52 was obtained. Although the E-field at the tip of the wire was reduced by 99.87% compared with the birdcage coil in this scenario, the global SAR of the 4-channel pTx was relatively low which resulted in an R value that was close to the FDA safety threshold.

**Table 2:** R: Ratio of 1g local SAR vs global SAR (FDA limit = 2.7).

Wire Position	Birdcage Coil	pTx 2	pTx 4	pTx 8
ρ = 2.5 cm, z = 0 cm	2.64x10^4^	0.127	2.52	0.472
ρ = 6 cm, z = 0 cm	6.88x10^4^	0.0189	0.437	0.0124
ρ = 7.5 cm, z = 0 cm	7.53x10^5^	0.0244	0.0266	0.0143
ρ = 8 cm, z = 2 cm	3.01x10^4^	0.327	0.326	0.243

**Fig 6:**
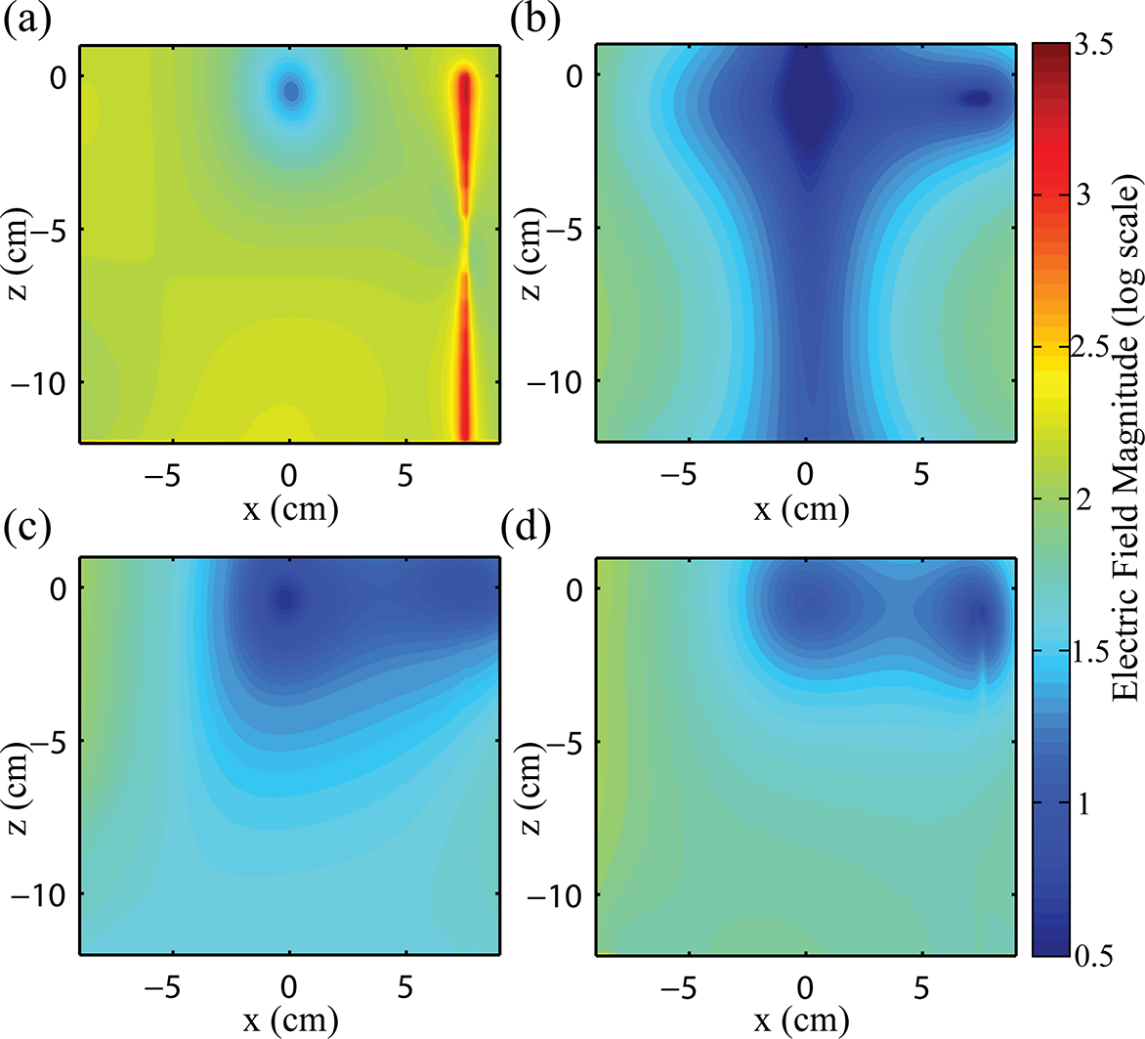
Contour plots of E-field magnitude. Contour plots of E-field magnitude on a log scale in the plane parallel to the wire for (a) 16-rung birdcage coil operating in quadrature mode, and optimized (b) 2-element pTx, (c) 4-element pTx and (d) 8-element pTx. All pTx configurations are operating with amplitudes and phase shifts determined for optimal E-field suppression (Table 1). Wire is located at x = 7.5 cm, z = -12 cm to z = 0 cm.

Fig 6 shows a contour plot of the E-field magnitude on a log scale for the different RF coil configurations, for a representative wire location (ρ = 7.5 cm, z = 0 cm). The plots show the E-field contours in the x-z plane, through the wire. As expected, RF transmission with the birdcage coil caused substantial elevation of the E-field along the wire in comparison to the E-field in the contralateral location, x = -7.5 cm (Fig 6A). The peak elevation in E-field occurred at both ends of the wire with an increase of approximately six-fold compared to the E-field at the contralateral location. The peak in E-field at the bottom of the phantom (z = -12 cm) was anticipated due to charge build up from termination of the wire at this location. In the clinical case, the wire would extend out of the field of view of the head coil and reports have shown that heating does not occur at this location [51]. Thus, the simulated result at z = -12 cm is not relevant and is not considered further. Considering RF transmission with each of the optimized pTx configurations in relation to the birdcage coil results, substantial E-field decrease was observed along the entire wire length (Fig 6B–6D). Improved E-field characteristics were also observed with increasing channel count, not only at the wire location but also in regions closer to the coil elements.

**Fig 7:**
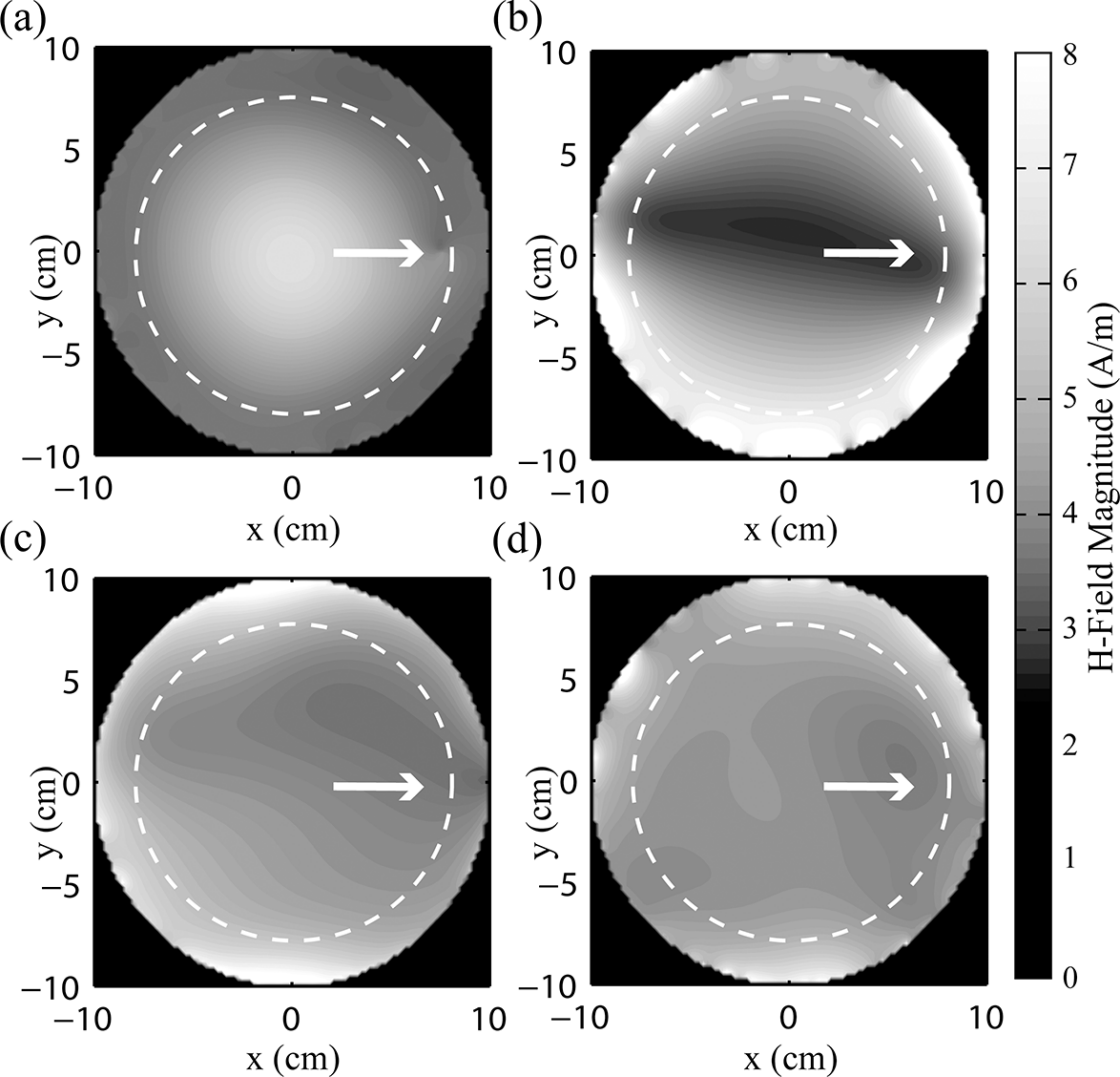
Magnetic field in the imaging plane (z = 0 cm). (a) Birdcage coil, signal variation = 24.3%, (b) 2-element pTx, signal variation = 46.0%, (c) 4-element pTx, signal variation = 17.3% and (d) 8-element pTx, signal variation = 9.0%. Wire is located at x = 7.5 cm, y = 0 cm, indicated by white arrow. Signal variation is the standard deviation divided by the mean calculated over circular region of interest with radius = 8 cm (dashed line).

Contour plots of B_1_-field magnitude in the imaging plane (x-y plane) are shown in Fig 7 with the wire tip at x = 7.5 cm, y = 0 cm (white arrow). As expected, the birdcage coil showed a field pattern with pronounced circular symmetry, and that included a small dipolar effect in the vicinity of the wire tip. The signal variation (standard deviation/mean, expressed as a percentage) of the birdcage coil was calculated as 24.3% over a circular region of interest (ROI) of ρ = 8 cm (dashed line). In comparison, the optimized 2-channel pTx configuration showed a marked decrease in B_1_-field magnitude along the axis containing the wire, extending laterally across the width of the medium, with a signal variation of 46.0%. However, the 4-channel and 8-channel pTx configurations provided substantially better B_1_ homogeneity, comparable to that of the birdcage coil (17.3% and 9.0%, respectively). Across the various wire locations, the magnetic field maintained this level of homogeneity.

**Fig 8:**
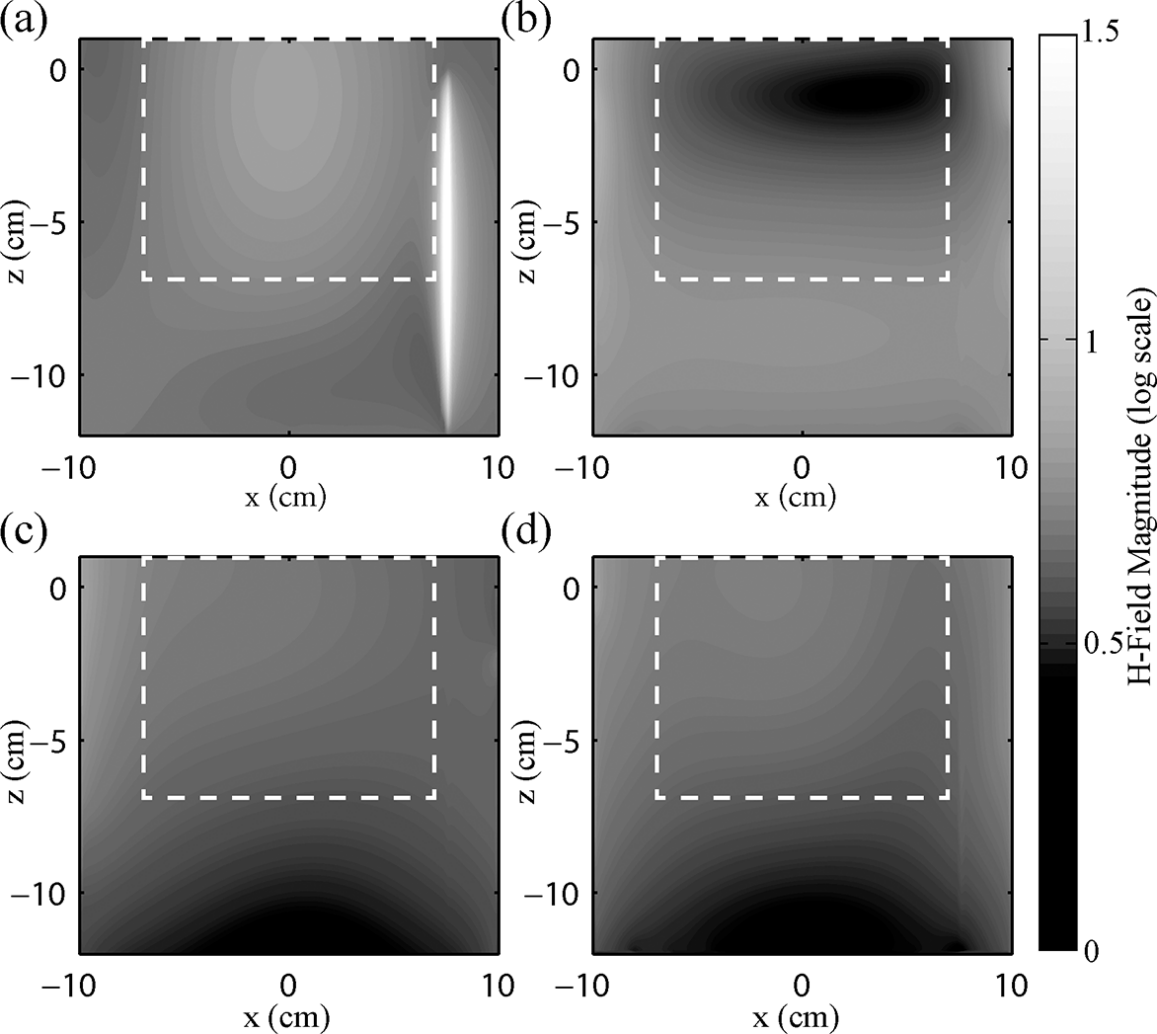
Contour plots of magnetic field magnitude. Contour plots of magnetic field magnitude on a log scale in the plane parallel to the wire for (a) 16-rung birdcage coil, signal variation = 17.1%, (b) 2-element pTx, signal variation = 42.1%, (c) 4-element pTx, signal variation = 13.9%, and (d) 8-element pTx, signal variation = 15.3%. Signal variation is the standard deviation divided by the mean calculated over a 14 cm x 8 cm rectangular region of interest (dashed line). The wire is located at x = 7.5 cm, z = -12 cm to z = 0 cm, and is readily identifiable in (a).

Fig 8 shows contour plots of magnetic field in the x-z plane, orthogonal to that shown in Fig 7, and with same position and orientation as the E-field simulation results shown in Fig 6. A large enhancement in the magnetic field was observed along the wire in the case of RF transmission with the birdcage coil (Fig 8A) to values up to 58 times the background levels in the image. To accommodate the large dynamic range of this effect, the magnetic field magnitude has been represented on a log scale. Because coupling between the wire and the E-field was significantly reduced when optimized pTx was used, negligible currents and associated magnetic fields were produced along the wire in all three pTx configurations. In the 2-element pTx configuration, the drop in magnetic field near the wire is seen in this orientation, producing an inhomogeneous field in the plane parallel to the wire. The 4- and 8-element pTx configurations show similar homogeneity to that of the birdcage coil, without the artifact created by currents in the wire. In a rectangular region of interest 14 cm x 8 cm (see Fig 8), the signal variation was 17.1%, 42.1%, 13.9% and 15.3% for the birdcage coil, 2-element, 4-element and 8-element pTx configurations, respectively.

When using optimized amplitudes and phase shifts, the E-field and H-field distribution remained approximately the same if the wire was omitted from simulation for all pTx configurations and wire locations, compared with the fields calculated when the wire was present. This is due to the negligible coupling that occurs between the RF fields and the wire when optimized amplitude and phase shifts were used.

### Experimental Verification

**Fig 9:**
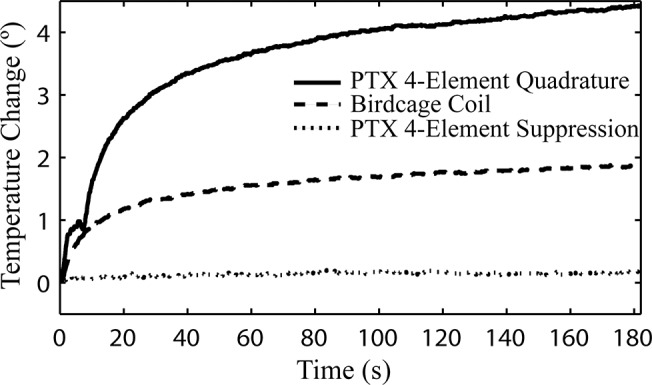
Change in temperature during FRFSE imaging of a uniform phantom containing an inserted copper wire. Results are shown for the 4-element pTx system operating in quadrature mode, in suppression mode, and the standard birdcage coil available with the MRI system.

Fig 9 shows plots of temperature change versus time, as recorded at the tip of a wire that was inserted in a cylindrical test phantom and subjected to MRI with a FRFSE sequence according to the experimental methods outlined above. Temperature was recorded for three scenarios: RF excitation with the standard transmit/receive 16-rung birdcage coil available on the MRI system; excitation with the 4-channel pTx coil in "quadrature mode", with phase shifts of 0°, 90°, 180°, and 270° applied to the coil elements to produce spatially homogeneous B_1_; and excitation with the 4-channel pTx coil in "suppression mode" with optimal phase shifts for reduced heating, as determined by simulation (φ_1_ = 40°, φ_2_ = 16°, φ_3_ = 200°, and φ_4_ = 301°). Whereas RF excitation with the birdcage coil produced a temperature increase of 2.0±0.15°C, and the pTx coil operating in quadrature mode produced an increase of 4.45±0.15°C, there was a negligible temperature increase of 0.2±0.15°C when the pTx coil was operated in suppression mode.

**Fig 10:**
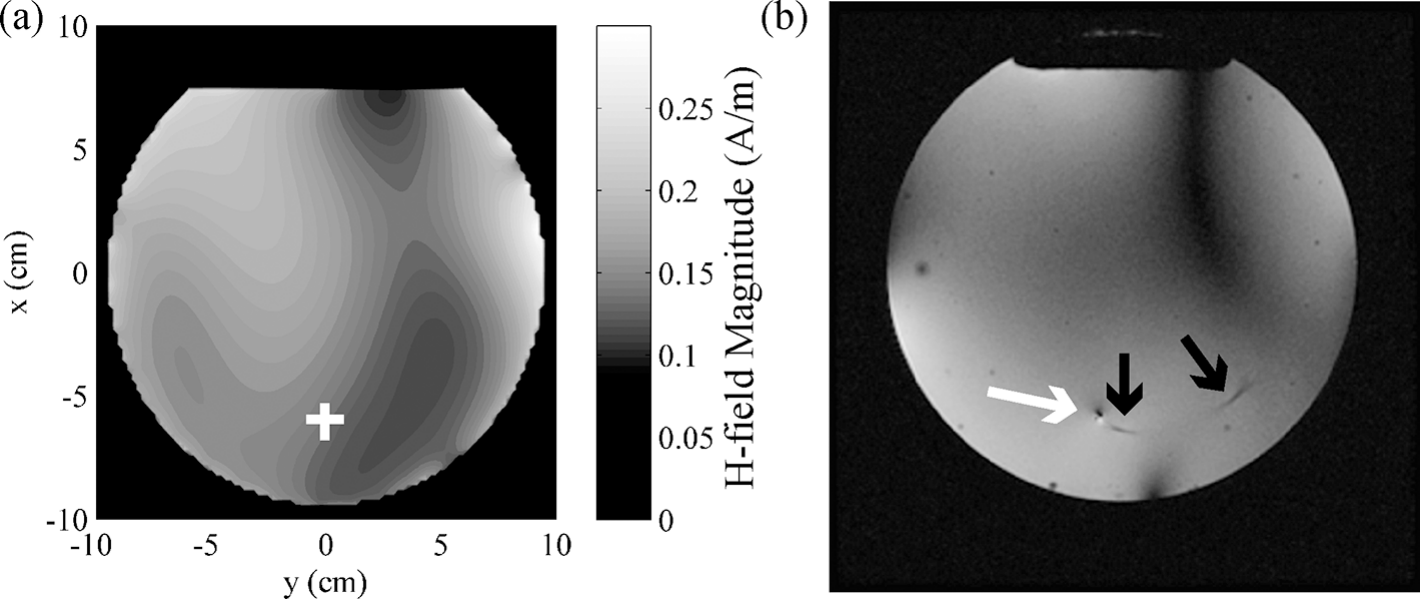
Axial slice in center of phantom obtained using 4-element pTx coil in suppression mode compared with simulation. (a) H-field obtained from simulation with optimal phase shimming (φ_1_ = 40.2°, φ_2_ = 15.7°, φ_3_ = 200.0°, φ_4_ = 301.1°). Wire position indicated by white cross. (b) Axial slice in center of phantom obtained with GRE sequence (φ_1_ = 40°, φ_2_ = 16°, φ_3_ = 200°, and φ_4_ = 301°). Wire position indicated by white arrow. Signal voids created by fiber optic temperature probe indicated by black arrows.

Fig 10 shows (a) the simulated H-field magnitude for the corresponding, optimal scenario (phase shifts of φ_1_ = 40.2°, φ_2_ = 15.7°, φ_3_ = 200.0°, φ_4_ = 301.1°), and (b) an axial image through the central plane of the phantom, acquired using a GRE sequence with the 4-channel pTx coil operating in suppression mode (phase shifts of φ_1_ = 40°, φ_2_ = 16°, φ_3_ = 200°, and φ_4_ = 301°). Signal intensity was reasonably uniform throughout the image, although a band of low signal intensity was observed between two coil elements in the upper right-hand quadrant, and various circular signal voids were observed due to the presence of air bubbles in the phantom material. The location of the wire tip was characterized by a small dipole artifact (white arrow), indicating that complete decoupling of the wire and RF field was not achieved (even though negligible temperature increase was observed in Fig 9). Additional signal void (black arrows) denoted the location of the fiber optic temperature sensor. These features notwithstanding, the signal intensity in the vicinity of the wire appeared to be sufficiently uniform for useful MR imaging.

## Discussion

Post-operative MRI of patients with DBS implants is severely restricted by regulatory agencies due to concerns of heating and tissue damage at the lead electrodes [52]. The present study has provided an early demonstration that with appropriately optimized static shimming of amplitude and phase, pTx can minimize RF coupling and subsequent heating in long, implanted leads. Thus, there is a basis for investigating this method further, toward future applications involving DBS patients. The simulation and experimental results that support this statement are discussed below, as well as issues that are likely worth investigating next.

In the present proof-of-concept study, pTx was optimized by simulation to suppress heating effects within a simple, uniform medium with an implanted, perfectly conducting wire. In this scenario, it was possible to suppress RF coupling and resultant E-field increases by at least 95% compared to excitation with a standard birdcage coil, while maintaining similar B_1_-field uniformity for pTx with high channel counts. The birdcage coil was chosen for comparison as presently it is the only RF source approved by the US Food and Drug Administration for imaging patients with DBS implanted devices (at 1.5 T).

Simulations were used to explore how suppression of RF coupling was affected by *N*, the number of channels used for pTx. For the channel counts investigated (*N* = 2, 4, 8), there was better E-field suppression and improved B_1_-field homogeneity with increasing *N* in all scenarios (different positions of the implanted wire). Although the E-field suppression was strong for all channel counts, a substantial increase in B_1_-field homogeneity was observed when progressing from 2-channel to 4-channel pTx systems, whereas the improvement from the 4-channel system to the 8-channel system was modest. In this simplified model, therefore, an argument can be made for *N* = 4 as the preferred channel count, from the standpoint of performance, as well as hardware cost and complexity. However, further investigation of effects such as tissue heterogeneity, as well as more complex pTx capabilities such as dynamic shimming, may ultimately influence this recommendation for future applications that involve DBS patients.

To provide suitable pTx RF inputs, a numerical optimization procedure was implemented with a cost function that included a term to minimize coupling between the RF field and the wire. Two cost functions were investigated with terms: (a) minimizing E-field at the wire tip and (b) minimizing *E*_*t*_ along the length of the wire (see Fig 1 and its related description). Cost function term (a) was aimed at minimizing the enhanced E-field at the tip of the wire as produced by RF coupling, whereas cost function term (b) was more rigorous, supporting solutions that minimized the E-field along the length of the wire.

Cost function (b) was investigated as part of the present work, but it was found to provide worse magnetic field homogeneity than cost function (a). One possible explanation for this finding is that cost function (a) provided more flexibility: by constraining the E-field only at the tip of the wire, multiple E-field distributions that produce minimal coupling were allowed, from which one or a subset provided acceptable B_1_ uniformity. In contrast, minimizing *E*_*t*_ along the wire also placed a substantial constraint on the associated B_1_ field that was produced. For example, minimizing the E-field at the tip of the wire still permits the polarity of *E*_*t*_ to alternate along the length of the wire in a manner that impedes current flow; minimizing *E*_*t*_ along the length of the wire eliminates these scenarios from consideration.

In this work, optimization of homogeneity of the magnetic field magnitude was used as a proxy for homogeneous MR signal. In reality, MR signal has a complicated dependence on multiple factors. Of particular interest to this work is the dependence of MR signal on the polarization of the magnetic field. Due to the direction of precession of spins excited in MR, transmit homogeneity is related to the right-hand circularly polarized component of the RF field (*B*_*1*_^*+*^) while the receive homogeneity is related to the left-hand circularly polarized component of the RF field (*B*_*1*_^*-*^). For a transmit/receive RF coil (such as the RF coil presented in this work), the MR signal (S) is related to *B*_*1*_^*+*^ and *B*_*1*_^*-*^[53]:

$$S\propto W\;\left|\text{sin}(I\left|B_{1}^{+}\right|\gamma \tau )\right|\left|B_{1}^{-}\right|$$ (3)

where *W* is a medium-dependent weighting factor, *I* is proportional to the current in the coil, γ is the gyromagnetic ratio and τ is the length of the B_1_ pulse. The approximation used in this work, $S\propto B_{1}^{+}\cdot B_{1}^{-}=\left|{\vec{B}}_{1}\right|$, is instructive to determine the ability to manipulate both E-field and B_1_-field concurrently. Further work is required to optimize the transmit field homogeneity, separately from the receive field homogeneity.

The optimal pTx solutions presented in this work also show that the E-field and B_1_-field magnitudes are not inextricably linked. In a particular plane of interest, static RF shim settings exist that cause the E-field to vary significantly in space (including regions of almost zero magnitude), whereas the associated B_1_-field remains relatively uniform. This is achievable due to the near-field nature of the problem. The current in the coil elements produces the B_1_-field, as described by Ampere’s law. The E-field is created by the change in charge distribution (which is related to the current) and follows Gauss’s law. The contribution of the B_1_-field to the E-field (and vice versa) through the Maxwell-Faraday law is substantially less than the contributions from current and charge oscillations in the coil element. Thus, though the fields are coupled through the relationship of current and charge distribution in the coils, the far field (or source-less) scenario where low E-field magnitude corresponds directly to low B_1_-field magnitude does not apply. This provides a theoretical basis for the technique and allows for safe imaging (negligible RF coupling and heating in the presence of conductive implants) while maintaining high imaging quality (uniform RF excitation).

The optimized static RF shim parameters (Table 1) also provide some additional insight into the mechanism of E-field suppression. The amplitude and phase shifts, together with the geometric contributions introduced by the position and orientation of each coil element surrounding the medium, cause the E-fields from each coil to interfere deconstructively at the wire tip. This effect creates the required condition to suppress RF coupling and maintain B_1_ homogeneity.

Experimental validation was performed to ensure that the E-field decrease obtained in simulation translates to temperature decrease in a practical, physical model. Practical implementation of the simulation described in this work was achieved with a relatively simple design. Inaccuracies in phase due to hardware constraints such as uncertainty in capacitor values and phase shifts introduced via the combiner/divider were not significant enough to invalidate the optimization. Multiple measurements were acquired experimentally with identical results observed, demonstrating the robustness of the design.

In the experimental validation of the numerical results discussed above, a 90% decrease in heating was observed at the tip of a copper wire inserted in a cylindrical, PAA phantom through excitation with a 4-channel pTx coil with optimal phase shifts applied to each channel (suppression mode) as compared with a transmit/receive birdcage coil. The temperature increase observed when RF excitation was undertaken with the pTx coil in suppression mode was negligible and on par with the experimental variability of the temperature probe. The temperature increase was also measured during transmission with the 4-channel pTx coil operating in quadrature mode (*ie*. with phase shifts of 0°, 90°, 180° and 270°) to demonstrate the potential for RF-induced localized heating with the custom-built coil. A 95% decrease in temperature measured at the tip of the wire was observed between the 4-channel coil operating in quadrature mode and in suppression mode. This underlines the effect of optimal phase shifts on coupling.

Similarly, promising image quality was obtained when optimized pTx was tested experimentally (Fig 10). Although image quality was reduced compared to a standard birdcage coil, and in particular a signal void was observed between two coil elements, substantial improvements can be made in future iterations. The pTx system used in the present work was built with priority placed on suppressing RF heating effects, with image quality as a secondary goal. In particular, the experimental 4-channel pTx system was built with RF splitting in transmit mode and RF combination in receive mode, using a passive splitter/combiner. This process reduces image quality because the MRI signals from different coil elements can destructively interfere. Future work will improve image quality through more sophisticated parallel receive techniques and image reconstruction [54]. In addition, it is anticipated that by including some dynamic RF shimming capability, in which coil elements can be driven by independent RF waveforms, image quality can be improved further.

As mentioned above, further investigations will be needed to study pTx capabilities in media that more closely resemble pertinent characteristics of the head, to move the technology closer to a clinical application. Such studies must also consider more pertinent models of the DBS implant itself. In the present work, the investigation of a bare, highly-conductive wire represents a problematic scenario for heating, as no insulation is used to reduce coupling and heat transfer into the surrounding tissue. When insulation is included, the E-field along the wire is reduced; however the E-field at the exposed tip is enhanced [55]. Optimized pTx can likely counteract this effect, but further dedicated investigations are needed. Investigations of realistic head models with varying tissue properties and perfusion are also needed. In this scenario, simulated temperature increases, calculated by the Pennes bioheat equation, will be required to link simulated E-fields and temperature increases measured in experiment. Lastly, optimization of the workflow for a clinical setting would improve the feasibility of this technique. Minimal reliance on large-scale simulation will improve clinical relevance and reduce pre-scan computation time. At this early stage, we suggest that B_1_-field maps (which may be measured in a pre-scan sequence) [56] and E-field maps (which can be calculated from the B_1_-field maps [57]), in conjunction with smaller-scale simulation, can potentially be used to determine input amplitude and phase to reduce computation time. These ideas will require further detailed investigation.

## Conclusions

The present work has demonstrated the feasibility of pTx with static RF shim settings optimized to suppress heating in long, implanted leads, as would be required for safe MRI of patients with DBS implants, while maintaining transmit B_1_ homogeneity for adequate imaging. Much additional work will be required in the future to prepare a practical clinical implementation of this technology, but the initial simulation and experimental results are promising.
